# Clinical and epidemiological characteristics of KPC-producing *Klebsiella pneumoniae* from bloodstream infections in a tertiary referral center in Italy

**DOI:** 10.1186/s12879-019-4268-9

**Published:** 2019-07-12

**Authors:** Lucia Brescini, Gianluca Morroni, Chiara Valeriani, Sefora Castelletti, Marina Mingoia, Serena Simoni, Annamaria Masucci, Roberto Montalti, Marco Vivarelli, Andrea Giacometti, Francesco Barchiesi

**Affiliations:** 1 Clinica Malattie Infettive, Dipartimento di Scienze Biomediche e Sanità Pubblica, Università Politecnica delle Marche, Azienda Ospedaliero-Universitaria Ospedali Riuniti Umberto I°-Lancisi-Salesi, Via Conca, 60126 Ancona, Italy; 2 Unità di Microbiologia, Dipartimento di Scienze Biomediche e Sanità Pubblica, Università Politecnica delle Marche, Via Conca, 60126 Ancona, Italy; 3 Laboratorio di Microbiologia, Azienda Ospedaliero-Universitaria Ospedali Riuniti Umberto I°-Lancisi-Salesi, Ancona, Italy; 4 Chirurgia Epatobiliare e dei Trapianti, Università Politecnica delle Marche, Azienda Ospedaliero-Universitaria, Ospedali Riuniti Umbero I°-Lancisi-Salesi, Ancona, Italy; 5 Malattie Infettive, Azienda Ospedaliera Ospedali Riuniti Marche Nord, Pesaro, Italy

**Keywords:** Bloodstream infections, *Klebsiella pneumoniae*, KPC, Colistin resistance

## Abstract

**Background:**

Bloodstream infections (BSI) due to *Klebsiella pneumoniae* carbapenemase (KPC)-producing *K. pneumoniae* (KPC-Kp) have become an important problem and they are associated with a high mortality rate. The aim of our study was to evaluate the clinical and epidemiological characteristics of KPC-Kp from BSIs.

**Methods:**

In this retrospective cohort study, conducted in a tertiary referral center in Italy, 112 patients with KPC-Kp BSIs diagnosed between February 2011 and December 2015 were identified. We evaluated the mortality at 30 days from the first positive blood culture. Survivor and non-survivor subgroups were compared to identify predictors of mortality.

**Results:**

The overall crude mortality was 35%. APACHE II score ≥ 15, septic shock at BSI onset, immunosuppressive therapy during the 30 days before the BSI onset, and the lack of a combination therapy with at least 2 active drugs emerged as independent predictors of mortality. Excluding patients with inadequate therapy, the mortality decreased to 25% while an APACHE II score ≥ 15 and the presence of septic shock remained independently associated with a negative outcome.

Two different pulsotypes were identified: pulsotype A belonged to ST512 and carried KPC-3 and pulsotype B belonged to ST307 and carried KPC-2.

**Conclusions:**

This study confirmed a high mortality rate of KPC-Kp BSIs. The outcome is heavily influenced by the patient’s clinical conditions. A therapeutic approach including a combination with at least two active drugs in vitro can improve the prognosis, unless patients received an appropriate therapy.

## Background

Bloodstream infections (BSIs) due to *Klebsiella pneumoniae* carbapenemase (KPC)-producing *K.pneumoniae* (KPC-Kp) have become an important problem in the last few years [1–3]. Infections caused by KPC-Kp isolates are associated with a high mortality rate, ranging from 22 to 72%. This variability depends on the different characteristics of the populations considered in the studies [2–8].

KPC-Kp strains are endemic in some countries, mainly in the United States, Israel, Latin American countries and Southern Europe [9]. The Annual Report of the European Antibiotic Surveillance Network (EARS-Net), published in 2016, reported a mean percentage of carbapenems resistance equal to 6,1%, with prevalent distribution in Greece, Italy and Romania [10].

Although several studies have demonstrated the efficacy of combination regimens in terms of decreased mortality, an effective treatment is still a challenge for clinicians [9]. Colistin, tigecycline and gentamicin were the most widely used antibiotics in these therapeutic regimens but increasing resistance to these drugs has been reported in recent years [11]. In particular, the emergence of colistin resistance among KPC-Kp is becoming a major problem. Literature data suggest an increasing mortality in patients infected with colistin-resistant (Col-R) KPC-Kp [12–15].

The aim of our retrospective cohort study was to evaluate the clinical and epidemiological characteristics of KPC-producing *Klebsiella pneumoniae* from bloodstream infections in a tertiary referral center in Italy.

In particular, the mortality at 30 days from the first positive blood culture was considered. Survivor and non-survivor subgroups were compared to identify predictors of mortality.

## Methods

### Study setting, data collection and definitions

The setting was a 980 beds Regional University Hospital in Ancona, Central Italy, including five intensive care units (ICUs), 11 medical and 11 surgical wards. All patients (≥18 years old) with BSIs due to KPC-Kp diagnosed between February 2011 and December 2015 were considered. A KPC-Kp BSI was documented by either one or more positive blood culture [16]. BSIs were defined as hospital-acquired if the index blood culture had been collected > 48 h after hospital admission and no signs or symptoms of infection had been noted at admission.

Patient variables included age, sex, presence of acute or chronic comorbidities, Charlson comorbidity index and APACHE II score, previous surgery (≤30 days before BSI onset), any invasive procedures (≤72 h before BSI onset), steroid and/or immunosuppressive therapy taken over the previous 30 days, previous antimicrobial therapy (≤30 days). The isolation of KPC-strains from other sites as well previous (≤30 days) or concomitant infections were also considered.

Hospitalization variables included nosocomial or healthcare-related infections, ward submitting index culture, time interval from admission, total days of hospitalization in the previous year.

Treatment variables included empiric antibiotic therapy, defined as treatment administered the same day of first positive blood culture collection (adequate or not, based on subsequent in vitro results), and post-antibiogram therapy (type and number of drugs [i.e.: monotherapy or combination therapy]). Antibiotic therapy was considered adequate if it met two criteria: drug administration within 5 days from BSI onset and the use of at least one drug which was active in vitro. An antibiotic was considered active in vitro when the isolate was susceptible to this specific drug.

The outcome measured was death within 30 days from the first positive blood culture. Survivor and non-survivor subgroups were compared to identify predictors of mortality. Patients who died within 24 h after the blood culture collection were excluded from the analysis.

### Microbiology

Identification of species was performed with MALDI-TOF mass spectrometry. Rectal swabs from all patients were collected at admission and screened for the presence of ertapenem-resistant enteric bacteria and main carbapenemases genes. Detection of KPC was assessed with Genexpert (Cepheid, Sunnyvale, CA, USA). KPC-Kp isolates were genotyped by XbaI-PFGE as previously described [17] and chromosomal DNA restriction patterns were interpreted following the criteria of Tenover et al. [18]. Some representative KPC-Kp isolates for each different PFGE profiles were subjected to MLST analysis [19] and amplification followed by Sanger sequencing of *bla*KPC genes. The allele sequences and sequence types (STs) were identified at http://bigsdb.pasteur.fr/klebsiella/klebsiella.html.

Susceptibility testing were performed by Vitek 2 system (bio-Merieux, Marcy l’Etoile, France). Colistin MICs were determined by the reference broth microdilution method and results were interpreted according to the EUCAST definition [20].

### Statistical analysis

Categorical variables were expressed as absolute numbers and their relative frequencies; continuous variables were expressed as median and interquartile range (IQR). Categorical variables were compared by the χ ^2^ or Fisher exact test, while continuous variables were evaluated by the Student *t* test (for normally distributed variables) or the Mann-Whitney *U* test (for nonnormally distributed variables). Variables which reached a statistical significance (*p* < 0.05) at univariate analysis were analyzed by multivariate logistic regression analysis to identify independent risk factors for mortality. Kaplan-Meier curves were constructed to compare 30-day survival between patients treated with at least 2 active drugs and those treated with < 2 active drugs and analyzed by log-rank test. The results obtained were analyzed using the software package SPSS 20.0 (IBM, Armonk, NY, USA).

## Results

During the study period, a total of 112 patients with KPC-Kp BSIs were identified. Baseline characteristics of the study cohort are reported in Table 1. The majority were male (72%) and had a low performance status (i.e.: 83% had Charlson Comorbidity Index ≥3 and 50% had an Apache II score ≥ 15). Cardiovascular and neurological diseases along with solid tumors were the most common underlying diseases (54, 34, 33%, respectively). Septic shock was present in 36% of the overall population at the BSI onset. The highest proportion of patients was hospitalized in ICUs (40%). Almost all patients (88%) carried a central venous catheter (CVC). A high proportion of patients was undergoing steroid or immunosuppressive therapies. Gastrointestinal surgery was the most common type of intervention characterizing these patients.

**Table 1:** Demographic and clinical characteristics of the study cohort

Variables	30-days outcome			
	All, 112	Death, 39 (35%)	Survive, 73 (65%)	*p*-value
Patients variables				
Sex				
• Male	80 (72%)	27 (69%)	53 (73%)	0.875
• Female	32 (28%)	12 (31%)	20 (27%)	
Age (years) median (IQR)	68 (55–76)	72 (58–78)	65 (54–75)	0.528
Charlsons Comorbidity Index ≥3	93 (83%)	37 (95%)	56 (77%)	0.030
Comorbidities				
• Diabetes	31 (28%)	9 (23%)	22 (30%)	0.566
• COPD	18 (16%)	7 (18%)	11 (15%)	0.900
• Haematological malignancies	16 (14%)	9 (23%)	7 (10%)	0.097
• Solid tumors	37 (33%)	15 (39%)	22 (30%)	0.496
• Chronic Hepatitis	10 (9%)	5 (13%)	5 (7%)	0.313
• Cardiovascular disease	66 (54%)	23 (59%)	43 (59%)	0.994
• Neurological disease	38 (34%)	6 (15%)	32 (44%)	0.005
• Chronic kidney disease	32 (29%)	14 (36%)	18 (25%)	0.301
• HIV	1 (1%)	0	1 (1%)	1.000
• Neutropenia	12 (11%)	8 (21%)	4 (6%)	0.033
• Gastrointestinal disease	23 (21%)	5 (13%)	18 (25%)	0.218
• SOT	5 (5%)	3 (8%)	2 (3%)	0.226
• Others	21 (19%)	5 (13%)	16 (22%)	0.357
Apache II score ≥ 15	56 (50%)	32 (82%)	24 (33%)	< 0.001
Acute comorbidities				
• Septic shock	40 (36%)	26 (67%)	14 (19%)	< 0.001
• pneumonia	48 (43%)	21 (54%)	27 (37%)	0.129
• Acute kidney failure	18 (16%)	8 (20%)	10 (14%)	0.506
• Gastrointestinal perforation	5 (5%)	4 (10%)	1 (1%)	0.049
• Trauma	10 (9%)	2 (5%)	8 (11%)	0.489
• Others	53 (47%)	16 (41%)	37 (51%)	0.329
Hospitalization variables				
Nosocomial infection	104 (93%)	36 (92%)	68 (93%)	1.000
Healthcare-related infection^a^	8 (7%)	3 (8%)	5 (7%)	
Wards submitting index culture				
• Intensive care unit	45 (40%)	15 (38%)	30 (41%)	0.945
• Surgery	27 (24%)	7 (18%)	20 (27%)	0.378
• Medicine	32 (29%)	14 (36%)	18 (25%)	0.301
• Other health care facilities	8 (7%)	3 (8%)	5 (7%)	1.000
Time interval (days) from admission, median (IQR)	23,5 (11–39)	23,5 (11–39)	24,5 (11,5–39)	0.541
Total previous hospitalization, median (IQR)^b^	30,5 (15–59.25)	33 (15–66)	27 (14.5–51.5)	0.352
Pre-infection variables				
Central venous catheter	99 (88%)	39 (100%)	60 (82%)	0.004
Other devices	102 (91%)	38 (97%)	64 (88%)	0.161
CVVH	8 (7%)	3 (8%)	5 (7%)	1.000
Invasive procedures^c^	31 (28%)	17 (44%)	14 (19%)	0.011
Steroid therapy^d^	43 (38%)	18 (46%)	25 (34%)	0.303
Immunosuppressive therapy^d,e^	29 (26%)	15 (39%)	14 (19%)	0.046
Previous Surgery^f^	60 (54%)	22 (56%)	51 (70%)	0.090
• Gastrointestinal surgery	30 (27%)	11 (28%)	19 (26%)	0.981
• Cardiovascular surgery	9 (8%)	5 (13%)	4 (6)	0.272
• Urologic surgery	7 (6%)	3 (8%)	4 (6%)	0.693
• Neurosurgery	13 (12%)	2 (5%)	11 (15%)	0.214
• Orthopedic surgery	9 (8%)	1 (3%)	8 (11%)	0.158
• Plastic surgery	3 (3%)	0	3 (4%)	0.550
• Thoracic surgery	3 (3%)	1 (3%)	2 (3%)	1.000
Microbiologic variables				
KPC rectal swab^g^	32 (29%)	16 (41%)	16 (22%)	0.056
Isolation of KPC from other sites				
• Urinary tract	30 (27%)	7 (18%)	23 (32%)	0.187
• Bronchial / pleural fluid	36 (32%)	11 (28%)	25 (34%)	0.660
• abdominal fluid	13 (9%)	6 (15%)	7 (10%)	0.370
• wounds	15 (13%)	6 (15%)	9 (12%)	0.872
Other infections, n° (%)				
• Previous infections^h^	35 (31%)	11 (28%)	24 (33%)	0.769
• Concomitant^i^	26 (23%)	10 (26%)	16 (22%)	0.834
Treatment variables				
Previous antibiotic therapy^d^	91 (81%)	33 (85%)	58 (80%)	0.680
• Penicillins	34 (30%)	12 (31%)	22 (30%)	1.000
• Cephalosporins	8 (7%)	1 (3%)	7 (10%)	0.258
• Carbapenems	51 (46%)	20 (51%)	31 (43%)	0.488
• Fluoroquinolones	32 (29%)	12 (31%)	20 (27%)	0.875
• Macrolides	4 (4%)	0	4 (6%)	0.296
• Aminoglycosides	10 (9%)	1 (1%)	9 (12%)	0.161
• Tigecycline	21 (19%)	8 (21%)	13 (18%)	0.924
• Colistin	14 (13)	6 (15)	8 (11%)	0.708
• Others	47 (42%)	19 (48%)	28 (38%)	0.391
Adequate empiric antibiotic treatment^j^	15 (13%)	6 (15%)	9 (12%)	0.872
Post-antibiogram therapy				
• Colistin-including therapy	44 (39%)	11 (28%)	33 (45%)	0.121
• Tigecycline-including therapy	57 (51%)	16 (41%)	41 (56%)	0.184
• Gentamicin-including therapy	46 (41%)	9 (23%)	37 (51%)	0.009
• Monotherapy	13 (12%)	3 (8%)	10 (14%)	0.537
• Combination therapy	89 (80%)	29 (74%)	60 (82%)	0.328
• Two-drug combinations	24 (21%)	10 (26%)	14 (19%)	0.581
• Combinations with ≥ three drugs	66 (59%)	19 (49%)	47 (64%)	0.160
• Carbapenem-excluding combinations	41 (37%)	16 (41%)	25 (34%)	0.615
• Carbapenem-including combinations	71 (63%)	23 (59%)	48 (66%)	
• Double-carbapenem combinations	5 (5%)	2 (5%)	3 (4%)	1.000
• Rifampin addition to combination	3 (3%)	2 (5%)	1 (1%)	0.240
• Adequate antibiotic treatment^j^	84 (74%)	21 (54%)	63 (85%)	< 0.001
-with one active drug	49 (44%)	17 (44%)	32 (44%)	1.000
-with two or three active drugs	35 (31%)	4 (10%)	31 (42%)	< 0.001
KPC-K isolate characteristics				
Colistin resistant	66 (59%)	22 (56%)	44 (60%)	0.846
Tigecycline resistant^k^	27 (37%)	10 (45%)	17 (33%)	0.436
Gentamicin resistant	14 (13%)	5 (13%)	9 (12%)	1.000
Amikacin resistant	97 (86%)	33 (85%)	64 (88%)	0.872
Fosfomycin resistant	72 (64%)	19 (49%)	53 (73%)	0.188
Bactrim resistant	92 (82%)	30 (77%)	62 (85%)	0.426
Meropenem MIC ≤8	4 (4%)	3 (8%)	1 (1%)	0.086
Meropenem MIC≥16	108 (96%)	36 (87%)	72 (99%)	
Pulsotype A – ST512^l^	97 (91%)	33 (89%)	64 (91%)	0.705
Pulsotype B – ST307^l^	10 (9%)	4 (11%)	6 (9%)	

Data are expressed as No. (%) unless otherwise specified

Abbreviations: *APACHE* Acute Physiology and Chronic Health Evaluation, *IQR* interquartile range, *COPD* Chronic obstructive pulmonary disease, *SOT* solid organ transplantation, *CVVH* Continuous Veno-Venous Hemofiltration

^a^Healthcare-associated infections are defined as described by Friedman, N. D. 2002. Health Care–Associated Bloodstream Infections in Adults: A Reason To Change the Accepted Definition of Community-Acquired Infections. Annals of Internal Medicine, 137 (10), 791. doi:10.7326/0003-4819-137-10-200211190-00007

^b^During the 12 months preceding BSI onset

^c^During the 72 h preceding BSI onset

^d^During the 30 days preceding BSI onset

^e^Excluding therapy with steroids

^f^During the 3 months preceding BSI onset

^g^The majority of rectal swabs was collected before the onset of KPC-Kp BSI, during of the same hospitalization

^h^During the 30 days preceding BSI onset. Previous infections included: lung infections (*n* = 16), BSIs (*n* = 5), SSTIs (*n* = 3), CNS infections (*n* = 2), abdominal infections (*n* = 2), UTIs (*n* = 2), abdominal infection plus BSI (*n* = 1), UTI plus BSI (*n* = 1), lung infection plus BSI (*n* = 1), lung infection plus SSTI (*n* = 1), lung infection plus UTI (*n* = 1)

^i^Concomitant infections included lung infections (*n* = 8), BSIs (*n* = 4), UTIs (*n* = 4), SSTIs (*n* = 2), abdominal infection (*n* = 1), BSIs plus lung infections (*n* = 3), BSI plus lung infection plus CNS infection plus SSTI (*n* = 1), abdominal infection plus BSI (*n* = 1), lung infection plus UTI (*n* = 1) and BSI plus UTI (*n* = 1). 10 BSI were polymicrobial

^j^Adequate therapy is defined as the use of at least one drugs to which the isolate was susceptible in vitro

^k^Strains showing tigecycline MIC ≥4 mg/L were considered non susceptible as stated by Marchaim et al. 2014. Major variation in MICs of tigecycline in Gram-negative bacilli as a function of testing method. J Clin Microbiol, 52:1617–21. doi:10.1128/JCM.00001-14

^l^Percentage of these variables are calculated only on the 107 patients from which bacterial strains were recovered

Ninety-six % of KPC-Kp demonstrated full resistance to meropenem (MICs ≥16 μg/ml) while the other strains (4%) were meropenem non-susceptible showing MICs of 8 μg/ml. Resistance rates of amikacin and trimethoprim-sulfamethoxazole were also high (86 and 82%, respectively) while gentamicin was the most active in vitro antibiotic (13% of isolates were resistant). Notably, resistance to colistin was found in 59% of the isolates. Colistin-resistant characterized all strains (100%) isolated in 2011 and 2012 while it decreased in the following years (47% in 2013 and 2014 and 50% in 2015).

Previous carbapenem therapy occurred in 46% of the patients. Empiric antibiotic treatment was considered adequate only in 13% of the overall population. Post-antibiogram therapy consisted of a combination regimen in 80% of the cases with a carbapenem-including approach being the most frequently utilized. Antibiotic treatment was considered adequate in 74% of the patients: therapy was administered with only one active drug or with two or three active drugs in 44 and 31% of cases, respectively.

Thirty-day mortality rate was 35%. A significantly high proportion of patients dying within 30 days had a Charlson Comorbidity Index ≥3, an APACHE II score ≥ 15, neutropenia, septic shock, gastrointestinal perforation, carried a CVC, underwent an invasive procedure within 72 h and received immunosuppressive therapy (p ranging from < 0.001 to 0.049). There was a trend, although no statistically significant (*p* = 0.056), of positive KPC rectal swabs in non-survival patients, collected before the BSI onset. On the contrary, survivors most frequently received combination therapy with at least two active drugs (*p* < 0.001) and gentamicin-including regimens in the postantibiogram phase (*p* = 0.009).

In the multivariate logistic regression analysis, APACHE II score ≥ 15, septic shock at BSI onset, immunosuppressive therapy during the 30 previous days, and the lack of a combination therapy with at least 2 active drugs emerged as independent predictors of mortality (Table 2).

**Table 2:** Multivariate analysis of risk factors for mortality in the study cohort

Variable	Adjusted OR (95% CI)	*p*
All patients (*n* = 112)		
Apache score II ≥15	12.260 (3.653–41.146)	< 0.001
Septic shock	6.542 (2.209–19.374)	0.001
Immunosoppressive therapy	4.363 (1.201–15.858)	0.025
Antibiotic treatment with < 2 active drugs	5.063 (1.382–18.546)	0.014
Adequate therapy (*n* = 84)		
APACHE II score ≥ 15	30.241 (4.163–219.700)	0.001
Septic shock	6.043 (1.096–33.303)	0.039
Hospitalization in internal medicine wards	7.299 (1.174–45.376)	0.033

**Fig. 1:**
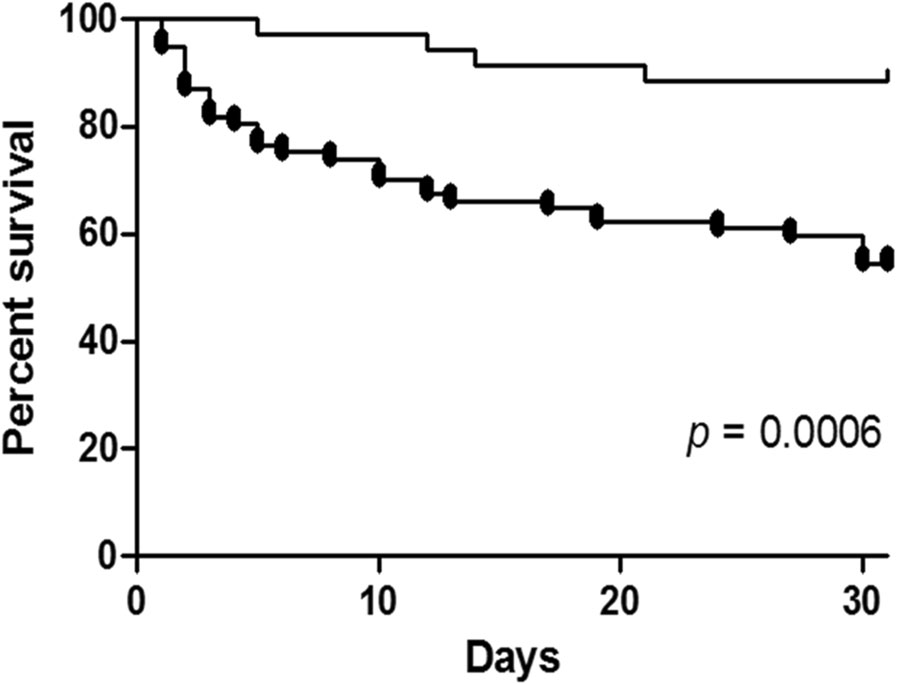
Kaplan Meier survival curves of patients treated with ≥2 active drugs (flat line) vs those patients treated with < 2 active drugs (dots line)

Survival curves confirmed the reduced mortality risk associated with a combination regimen containing at least 2 active drugs compared to other groups of treatment (*p* = 0.0006, Fig. 1).

We performed a further analysis by excluding those patients (*n* = 28) not having received an adequate therapy. This subgroup of patients showed a 30-day mortality of 25%. Independent risk factors for mortality were APACHE II score ≥ 15 (OR 30.241; 95% CI, 4.163 to 219.700; *p* = 0.001), septic shock (OR 6.043; 95% CI, 1.096 to 33.303; *p* = 0.039) and hospitalization in internal medicine wards (OR 7.299; 95% CI, 1.174 to 45.376; *p* = 0.033).

A total of 107 out of 112 isolates were molecularly identified. XbaI-PFGE disclosed two different pulsotypes: the most common was the A pulsotype (97/107 isolates) while B pulsotype was less diffused (10/107 isolates). Ten representative strains from pulsotype A underwent MLST and all belonged to ST512 and harboured KPC-3, differently the 4 representative isolates with pulsotype B that belonged to ST307 and carried KPC-2. Notably, ST307 strains were all susceptible to colistin. We did not find any correlation between 30-day mortality and sequence type.

## Discussion

In this study we evaluated 30-day mortality in patients with BSIs due to KPC-Kp. We found a crude mortality of 35%. These data are consistent with previous reports, in which the mortality rate ranged from 22 to 72% [3, 4, 7, 8]. This wide variation in mortality can be due to several factors including patient characteristics, type and timing of antibacterial therapy as well resistance patterns of the isolates. In the present study, in which up to 90% of the isolates had meropenem MIC > 16 μg/ml, we did not find any beneficial effect of a carbapenem-containing regimen on survival. Our data agreed with those reported in the literature suggesting that efficacy of meropenem-containing regimens decreased when meropenem MICs were > 8 μg/ml [2–4].

One study evaluated the achievement of pharmacokinetic/pharmacodynamics (PK/PD) targets of meropenem, administered as an extended 3-h infusion of 2 g every 8 h in 19 critically-ill patients with KPC-Kp BSIs. As expected, PK/PD target was not achieved when meropenem MICs ranged from 256 to 1024 μg/ml [21]. On the other hand, one recent study showed that high-dose continuous infusion of meropenem, in combination with other antimicrobials with activity against Gram-negative bacteria, was effective in the treatment of KPC-Kp infections caused by isolates with meropenem MICs ≥64 μg/ml [22]. Overall, these data indicate that there are still conflicting results on the use of carbapenem-containing regimens when meropenem MIC is high. The recent introduction of new antimicrobial therapies (i.e.: 3rd generation cephalosporin/carbapanems associated with innovative lactamase inhibitors) will overcame this issue.

We found an extremely high resistance rate to colistin (59%). However, we did not observe a difference in mortality between patients infected with colistin-susceptible and Col-R isolates. Several studies showed a relationship between Col-R isolates and mortality [12, 15]. In particular, a multicenter, retrospective study conducted in six Italian hospitals, showed a colistin resistance equal to 20% [12]. Mortality rate in this subgroup of patients was significantly higher than that reported for patients infected with colistin-susceptible isolates (51% vs 39%, respectively). The same study also noted an increasing prevalence over time of Col-R KPC-Kp and an association between colistin resistance and previous colistin therapy [12]. In contrast with this findings and other data available in the literature [2, 11, 12, 15], we found a decrease of Col-R KPC-Kp isolates during the study period. This phenomenon can be due to, at least, two factors: the appearance of a new clone susceptible to colistin in 2014 (ST307) and the progressive implementation in our center of the antimicrobial stewardship with significant reduction of colistin use in clinical practice.

Independent risk factors associated with increased mortality in our cohort of patients were an APACHE II score ≥ 15, the presence of septic shock, the immunosuppressive therapy, and the lack of a combination therapy with at least two active drugs. The first two factors were repeatedly reported in previous studies [2, 3, 13] and emphasize how the patient’s clinical status is of paramount importance in determining the outcome of the infection. Although the presence of a current immunosuppressive therapy theoretically correlates well with a negative outcome of the infection, one study found that an immunosuppression status unexpectedly correlated with a better outcome [23]. These paradoxical results have been explained by the exclusion of patients highly likely to have poor outcome (i.e.: too short antibiotic therapy [≤48 h] and the presence of polymicrobial bacteriemia). Other retrospective studies reported a positive association between combination therapy and 30-day survival [3, 4]. Although we found that the number of agents with activity in vitro (at least 2 active drugs) is an important factor to guarantee a positive outcome, contrarily to other studies, we did not observe any correlation between the type of therapeutic association and outcome [9]. A possible bias could include the fact that patients who live longer were more likely to received two active drugs. In order to minimize this effect, we performed a sub-analysis excluding patients that have not received an adequate therapy; the results obtained were slightly different. Indeed, while APACHE II score ≥ 15 and the presence of septic shock remained independent risk factors associated with increased mortality, hospitalization in internal medicine wards emerged as negative prognostic factor. Moreover, in this sub-group the combination therapy disappeared as a positive prognostic factor. These results may be due to the fact that patients received an appropriate drug, the outcome is heavily influenced by the patient’s clinical conditions as well as an intensive care unit stay might yield a more adequate support. The majority of KPC-Kp isolated in our hospital belonged to ST512 and produced KPC-3. This finding was not surprising taking into account that most of hospital-associated KPC-Kp recovered in Italy belongs to this clonal lineage as reported by a recent national study [24]. The other clone ST307 was already recovered in Italy [24] and belongs to a novel widespread lineage that could emerge as a new clinically relevant clone [25]. It’s interesting to note that in our center, KPC-Kp ST307 clone appeared in the early 2014 being isolated in a patient coming from a long-term care facility and it was responsible of a mini-outbreak in several wards. This finding emphasizes how hospital outbreaks could be supported by external clones reaching the hospital and highlights the importance of surveillance also in non-hospital structures.

The present study has some limitations. First, being a retrospective observational study our sample size is too small to allow us to detect subtle differences in treatment outcome. Second, since our data come from a single center experience, our findings may not be relevant to other patients population. Third, although we have made every attempt to collect and analyze as many as clinical data as possible to reveal useful information for patient management, some variables could not be explored because of missing data. Fourth, since EUCAST breakpoints are different from those of CLSI, some results may be different if the CLSI breakpoints are applied. Finally, our study did not include newer agents (e.g., ceftazidime-avibactam, meropenem-vaborbactam) and it is unknown whether combination therapy involving these agents would have been associated with improved outcomes.

## Conclusions

This study confirmed a high mortality rate of KPC-Kp BSIs. The outcome is heavily influenced by the patient’s clinical conditions. A therapeutic approach including a combination with at least two active drugs in vitro can improve the prognosis, unless patients received an appropriate therapy. Additional data are needed to further elucidate this finding especially in light of the introduction of the new molecules.

## Data Availability

The data that support the findings of this study are available from Azienda Ospedaliero-Universitaria Ospedali Riuniti Umberto I°-Lancisi-Salesi but restrictions apply to the availability of these data, which were used under license for the current study, and so are not publicly available. Data are however available from the authors upon reasonable request and with permission of the Institutional Review Board of Azienda Ospedaliero-Universitaria Ospedali Riuniti Umberto I°-Lancisi-Salesi.

## Abbreviations

APACHE: Acute Physiology and Chronic Health Evaluation
BSI: Blood stream infections
CI: Confidence interval
Col-R: Colistin resistant
COPD: Chronic obstructive pulmonary disease
CVC: Central venous catheter
CVVH: Continuous Veno-Venous Hemofiltration
HCC: Hepatocellular carcinoma
ICU: Intensive care unit
IQR: Interquartile range
KP: *Klebsiella pneumoniae*
KPC: *Klebsiella pneumoniae* carbapenemase
MALDI-TOF: Matrix Assisted Laser Desorption/Ionization Time of Flight Mass Spectrometry
MICs: Minimum inhibitory concentrations
SOT: Solid organ transplantation
SPSS: Statistical Package for the Social Sciences
SSTIs: Skin and soft tissue infections
UTIs: Urinary tract infections
